# Cystic Fibrosis Patients with F508del/Minimal Function Genotype: Laboratory and Nutritional Evaluations after One Year of Elexacaftor/Tezacaftor/Ivacaftor Treatment

**DOI:** 10.3390/jcm11236900

**Published:** 2022-11-22

**Authors:** Vincenzo Carnovale, Filippo Scialò, Monica Gelzo, Paola Iacotucci, Felice Amato, Federica Zarrilli, Assunta Celardo, Giuseppe Castaldo, Gaetano Corso

**Affiliations:** 1 Department of Translational Medical Science, University of Naples Federico II, 80131 Naples, Italy; 2 Department of Translational and Medical Science, University of Campania “L. Vanvitelli”, 80131 Naples, Italy; 3 CEINGE—Biotecnologie Avanzate, 80131 Naples, Italy; 4 Department of Molecular Medicine and Medical Biotechnology, University of Naples Federico II, 80131 Naples, Italy; 5 Department of Clinical Medicine and Surgery, University of Naples Federico II, 80131 Naples, Italy; 6 Department of Clinical and Experimental Medicine, University of Foggia, 71122 Foggia, Italy

**Keywords:** cystic fibrosis, CFTR modulators, lipid profile, protein metabolism, inflammation

## Abstract

The last ten years have been characterized by an enormous step forward in the therapy and management of patients with Cystic Fibrosis (CF), thanks to the development and combination of *Cystic Fibrosis Transmembrane Receptor* (*CFTR*) correctors and potentiators. Specifically, the last approved triple combination elexacaftor/tezacaftor/ivacaftor has been demonstrated to improve lung function in CF patients with both homozygous Phe508del and Phe508del/minimal function genotypes. Here we have assessed the effect of elexacaftor/tezacaftor/ivacaftor in patients carrying the Phe508del/minimal function genotype (*n* = 20) after one year of treatments on liver function and nutrient absorption with a focus on lipid metabolism. We show that weight, BMI, and albumin significantly increase, suggesting a positive impact of the treatment on nutrient absorption. Furthermore, cholesterol levels as a biomarker of lipid metabolism increased significantly after one year of treatment. Most importantly, we suggest that these results were not dependent on the diet composition, possibly indicating that the drug improves the hepatic synthesis and secretion of proteins and cholesterol.

## 1. Introduction

Cystic fibrosis (CF) is an autosomal recessive disease caused by mutations in the *Cystic Fibrosis Transmembrane Receptor* (*CFTR*) gene. *CFTR* malfunction causes an impaired transport of chloride and bicarbonate resulting in an accumulation of thick mucus that compromises respiratory, biliary, pancreatic, and gastrointestinal function, causing a plethora of clinical manifestations affecting patients with CF from birth [1].

Although lung disease is associated with higher mortality in patients with CF, the impaired digestion and malabsorption of food macro- and micro-nutrients (NM), despite a specific dietary regimen associated with digestive enzyme replacement therapy (ERT), cause a lipid imbalance and a prolonged inflammatory state which also represent specific distinctive signs of this pathology. In fact, they have a reduced liver secretion of lipoproteins with low levels of circulating cholesterol, and the evaluation of digestion and the analysis of absorption lipid biomarkers in CF patients would greatly help to manage these patients [2,3]. The hypocholesterolaemia was also observed in the mouse model of CF homozygous for the Phe508del *CFTR* mutation in the FVB/129 outbred background [4].

Despite the hundreds of different mutations that cause CF, more than 85% of affected people have at least one copy of *CFTR* with the Phe508del mutation, which significantly alters the processing, intracellular trafficking, and structural stability of the *CFTR* protein. Consequently, the defect greatly reduces the amount of protein on the apical membrane of epithelial cells. Furthermore, the *CFTR* Phe508del protein exhibits a channel alteration that further restricts the flux of anions [5,6,7,8].

Over the past 10 years, these molecular defects have been targeted for the purpose of restoring Phe508del *CFTR* protein function. Now, the development of new drugs, small molecules called potentiators and correctors, has completely revolutionized the therapy of patients with CF, from treating the symptoms to aiming to restore *CFTR* functions. These drugs are of great importance not only to study their effects on lung function but, as we described earlier, to assess their effects on nutrient absorption, and particularly, on lipid profile [2]. For example, in 2012 the FDA approved the potentiator Ivacaftor (IVA), and in 2015, the approved combination with the corrector lumacaftor (IVA/LUMA) was shown to improve lung function and reduce pulmonary exacerbations [9,10].

Nevertheless, none of the double combinations showed sufficient efficacy in CF patients who had a Phe508del allele and a mutated *CFTR* allele unresponsive to these modulators, which may be due to their inability to restore *CFTR* activity completely. These mutations are named “minimal function” (MF) [11,12]. Recently, we showed that although IVA/LUMA has some effects on cholesterol metabolism, this combination does not improve hypocholesterolemia in patients with CF [2].

Differently from IVA/LUMA, the recently FDA-approved triple combination of two correctors, elexacaftor/tezacaftor and a potentiator Ivacaftor (ETI) [13], has been described as a “silver bullet”, since it has been able to improve lung function in different clinical trials on CF patients with both homozygous Phe508del and heterozygous P/MF [14,15,16].

In this work, in addition to this knowledge, we sought to study in a cohort of CF patients carrying P/MF genotypes, treated with ETI, if nutrition absorption was improved after 3 months and one year of treatment. We have focused especially on the lipid profile, showing that after one year, ETI treatment is able to improve both albumin synthesis and secretion and increase the hepatic synthesis/secretion of lipoproteins, as evidenced by circulating non-HDL cholesterol.

## 2. Materials and Methods

### 2.1. Patients and Nutritional Data

In this study, the inclusion criteria were as follows: CF patients with heterozygous P/MF [14], age > 12 years, and severe lung disease. Therefore, we recruited a group of 20 CF patients with heterozygous P/MF who were starting the ETI therapy at the Regional Cystic Fibrosis Reference Center for adults (Department of Translational Medical Sciences, University of Naples Federico II). The Italian Drug Agency (AIFA) approved the treatment with ETI to correct the underlying cause of cystic fibrosis in patients at least 12 years old carrying the *CFTR* gene with a Phe508del mutation and a mutation with minimal function (see Table 1). The *CFTR* genotype of all patients was defined by first-level molecular analysis [17], and in all cases, gene sequencing excluded other ones in cis position [18,19]. All included patients had pancreatic insufficiency and all of them were treated with pancreatic enzyme replacement therapy. All patients were already receiving multivitamin supplementation (2 tabs of DKX, Neupharma, Imola, 40026 Italy) containing vitamin E (362 mg/day). The diet composition for cystic fibrosis patients follows the ESPEN-ESPGHAN-ECFS guidelines [20].

The diet of 12 patients enrolled in the present study was monitored by consumption for at least one week before starting therapy and after 12 months of therapy with ETI, following a procedure previously described [21]. Briefly, patients were instructed to complete a daily diary at the end of each meal and for a week. The quantity of each food was measured with a food-weighing scale or, if not possible, by using home measures (teaspoon, spoon, ladle). Food and drink were indicated in as much detail as possible [22]. The quantitative nutritional composition of the ingested macro- and micro-nutrients was calculated using a homemade calculator based on the tables published by the National Research Institute for Food and Nutrition (INRAN 2013, Italy). The study was performed according to the current version of the Helsinki Declaration, and all subjects were informed and gave written permission to process anonymously their clinical results for scientific aims. The study was approved by the Ethical Committee of the University Federico II of Naples.

### 2.2. Patient Stratification According to Genotype

All patients carried a Phe508del mutation allele and an MF mutation. We classified them in 3 groups according to Munck et al. [23], based on the type of MF mutation they carried in heterozygosity with the Phe508del. Briefly, the first group corresponds to “Truncation mutation”, where we included patients carrying an early stop truncation of *CFTR*; therefore, a complete absence of the protein. The second group corresponds to “Canonical Splice mutations, Frameshift mutations”, where we included patients carrying mutations of I class leading to a late stop truncation of *CFTR* and other MF defects. As Munck et al. did, in the third group we included patients with “Class II, III, IV mutations not responsive to ivacaftor or tezacaftor”.

### 2.3. Biochemical Analyses

Blood samples were collected from fasted patients before ETI treatment (baseline) and after 3 and 12 months of treatment. Serum samples were separated from blood cells and then analyzed for biochemical parameters by an automated biochemistry analyzer (Architect CI-16200 Integrated System, Abbott Diagnostics, Rome, Italy), as previously described [24,25]. All procedures were performed under internal and external laboratory quality control assurance. Serum vitamins A and E were analyzed by an isocratic high-performance liquid chromatography (HPLC) method (Vitamin A/E by HPLC assay, Bio-Rad Laboratories, Segrate, Italy) with an HPLC-UV system (Agilent 1260 Infinity Quaternary LC coupled with BIO-RAD UV-1806 detector, Bio-Rad Laboratories, Segrate, Italy).

### 2.4. Statistical Analysis

A Shapiro-Wilk test has been used to test the normality of data distributions. Continuous normal data were reported as mean (standard deviation, SD), while continuous non-parametric data were reported as median (interquartile range, IQR). For normal data, the comparisons of paired data among three groups were performed by one-way repeated measures ANOVA (RMA) and Bonferroni as post hoc test. The comparisons between two normal independent groups were performed by Student’s unpaired *t*-test. For non-parametric data, the comparisons of paired data among three groups were performed by Kruskal Wallis test and Wilcoxon signed-rank test for pairwise comparisons. Categorical data were reported as frequency (percentage) and the chi-square test was used to compare the frequency between groups. The correlations between variables were evaluated by Spearman’s rank correlation analysis. Statistical analyses were performed by SPSS (version 27, IBM SPSS Statistics, New York, NY, USA). The significance was accepted at the level of *p* < 0.05.

## 3. Results

The study population characteristics, including demographic, genetic, and clinical phenotypes at baseline, are shown in Table 1. The patients (*n* = 20) included eight males (40%) with a mean age of 31.9 years (range: 22–44 years). Regarding the genotype, the patients were divided into three groups according to Munck et al. [23] and as described in the material and methods. After 3 months of treatment with ETI, the mean (SD) of FEV1 increased significantly (*p* < 0.0001) from 31.4 (8.2)% to 42.3 (12.5)% (relative percentage change from baseline value as mean (SD): +36.1 (27.3)%), and after 12 months, it further increased to 45.7 (14.6)% (*p* < 0.0001) (relative percentage change from baseline value as mean (SD): +47.3 (35.0)%). All anthropometric and laboratory data collected from the 20 patients with CF at the three time points, i.e., at baseline and after 3 and 12 months from the start of ETI therapy, are shown in Table 2. In particular, we reported the evaluation of biomarkers of inflammation, glucose metabolism, lipid metabolism, and hepatobiliary injury/function. We found that both the median of weight (58.2 vs. 53.0 kg; *p* = 0.021) and body mass index (BMI) (23.1 vs. 20.0 kg/m^2^; *p* = 0.002) were significantly increased after one year of treatment. Moreover, C-reactive protein (CRP), a well-known marker of inflammation, was significantly decreased (0.30 vs. 0.71 mg/dL; *p* < 0.0001). Additionally, the CRP/albumin ratio was significantly decreased after one year of treatment (Table 2). After one year of treatment, the median of direct and total bilirubin increased from 0.24 to 0.33 mg/dL (*p* = 0007) and from 0.50 to 0.95 mg/dL (*p* = 0003), respectively, in addition to an increase in the median of aspartate aminotransferase (AST) from 20.0 to 27.5 U/L (*p* = 0.046). In Table 3 are reported the number of patients and the biomarkers whose values were upper limit at baseline and after one year of ETI treatment. More interesting is the significant increase in albumin levels (4.5 vs. 4.1 g/dL; *p* < 0.0001), an increment of the median of at least 10%. The assessment of lipid profile showed a significant increase in median of both total cholesterol (157 vs. 134 mg/dL; *p* = 0.035) and cholesterol of low-density lipoproteins (LDL) (91 vs. 47 mg/dL; *p* = 0.0001), while a decreasing trend of cholesterol of high-density lipoproteins (HDL) was observed (49 vs. 56 mg/dL; not significant, n.s.). In addition, we found a significant increase in non-HDL cholesterol (97.0 vs. 63.5; *p* = 0.004) and in LDL/HDL cholesterol ratio (1.93 vs. 0.69; *p* = 0.046). Among lipophilic vitamins, we observed a significant increase in vitamin A (47.8 vs. 28.1 µg/dL; *p* = 0.002) median together with an increasing trend of vitamin E (1316 vs. 1181 µg/dL; n.s.).

In addition, we compared all the evaluated biomarkers between CF patients with (*n =* 12) and without (*n =* 8) CF-associated liver disease (CFLD) at baseline, as well as after 3 and 12 months from the start of ETI therapy. We did not find significant differences at baseline, while after 12 months of therapy, we observed higher levels of serum CRP (median (IQR): 0.33 (0.33–0.85) mg/dL, *p* = 0.043) and insulin (median (IQR): 16.6 (11.7–64.0) mU/L; *p* = 0.003) in patients with CFLD than in those without CFLD (CRP < 0.33; insulin median (IQR): 4.3 (3.5–4.9) mU/L). Among the twelve patients with CFLD, eight (66.6%) patients had also CF-related diabetes (CFRD), and the comparison at the three time points between CF patients with and without CFRD gives comparable results.

Table 3 shows the comparison of the patient numbers with and without CFLD, and with altered serum CRP and liver parameters at baseline and after one year of ETI therapy. We observed that the number of patients with altered CRP and albumin levels decreased after the therapy in both groups, while we found an increased number of patients with altered liver parameters with a comparable grade for both patient groups.

Table 4 reports the comparison between the nutritional data at baseline and 12 months. No significant differences have been observed between the two time points. Furthermore, we wanted to investigate whether there were any relationships between the nutritional components and the biochemical markers measured in the serum of CF patients. The results of Spearman correlation analysis between the anthropometric and biochemical parameters that significantly differ after one year of ETI therapy and the nutritional data from 11 of 20 patients (at baseline and after 12 months of treatment) are reported in Tables S1 and S2. At baseline, we found a direct association between the diet carbohydrates (g) and serum vitamin A (µg/dL), and an inverse association of diet monounsaturated fatty acids (MuFA, g) versus serum LDL, non-HDL cholesterol, and LDL/HDL ratios (Table S1). After 12 months of treatment with ETI, we observed a direct association between diet proteins (g) and serum albumin (g/dL), while the diet cholesterol (g) was inversely correlated with the BMI (kg/m^2^). In addition, we found an inverse correlation between diet carbohydrates (g) versus non-HDL cholesterol, and LDL/HDL ratios (Table S2).

The results shown above highlight that, after one year of treatment with ETI, the patients with CF have an overall improvement in hepatic albumin synthesis and/or excretion. Additionally, considering that non-HDL cholesterol increases significantly and that it indirectly represents the overall hepatic synthesis of very low density lipoproteins (VLDL) and apoprotein B100, we wanted to investigate the relationship among both liver biomarkers and genotypes described in the three groups reported in Table 1.

**Figure 1:**
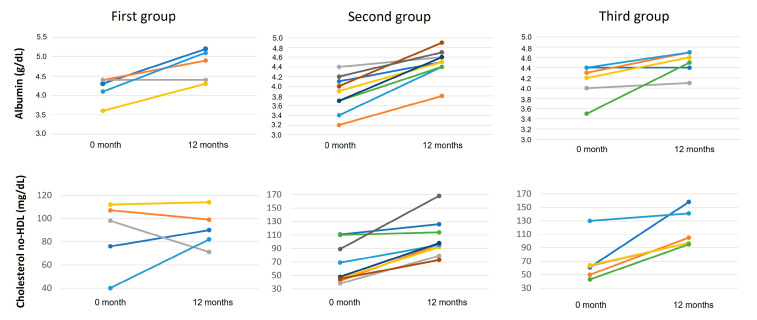
Serum albumin and non-HDL cholesterol trend in CF patients at baseline and 1 year of ETI therapy. The groups have been subdivided as described in Table 1.

Figure 1 shows the trends of serum albumin and non-HDL cholesterol levels in CF patients subdivided into the three genotype groups at baseline and after one year of ETI therapy (see Table 1). As shown in Table S3, after one year of ETI therapy, the albumin increased on average by 15%, 19%, and 11% in the first, second, and third groups, respectively, but these differences were not significant among the three genotypes groups, while the increase in non-HDL cholesterol was significantly (*p* < 0.05) lower in the first group (18%), compared with the second group (59%) and the third group (69%).

## 4. Discussion

In the last 10 years, the development of new drugs called modulators, such as potentiators and correctors, has opened a new era for about 90% of people with CF carrying a Phe508del mutation in the *CFTR* gene [26]. These compounds are small molecules that directly target *CFTR*, correcting its folding, membrane transport, and gating functions. Moreover, they have been combined to develop new therapies able to improve *CFTR* processing at different stages of its molecular processing [26,27]. These combinations, such as the corrector LUMA plus the potentiator IVA, improved lung function only slightly after the treatment. Regarding its metabolic effects, we have shown that it was unable to correct the hypocholesterolaemia in treated CF patients [2].

Although improvement in lung function is the first goal to be achieved in caring for these patients, CF is a multi-organ disease that includes the function of the hepato-biliary system, the gastrointestinal tract, and the pancreas, causing malabsorption of nutrients, an important aspect that these new therapies should aim to improve [28,29,30]. Therefore, it is of fundamental importance to study specific biomarkers to evaluate the function of the above organs in these patients.

Differently from IVA/LUMA, the new triple combination of two correctors, elexacaftor/tezacaftor, and IVA as a potentiator has been demonstrated to improve lung function in CF patients having both homozygous Phe508del and heterozygous P/MF genotype [31,32].

Here we have assessed the biochemical parameters with a focus on liver injury/function and lipid profile in 20 P/MF CF patients before starting ETI treatment, and after 3 and 12 months of therapy. Here we show that, after one year of treatment, parameters related to nutrient absorption such as weight, BMI, and albumin were all significantly increased, suggesting a better nutritional status. This was also mirrored by lipid metabolism with an evident median increase of about 18% of total cholesterol, which was not dependent on the dietetic regimen. This is an important result, demonstrating that, unlike IVA/LUMA, which was not able to correct hypocholesterolaemia, the triple combination ETI increased the serum level of cholesterol. However, it should also be considered that the total variability (biological and analytical) of this parameter is about 10% [33,34]; therefore, the increment observed in this study is clinically significant.

Furthermore, the data showed a significant reduction in serum CRP (−57.7%), suggesting a decrease in systemic inflammation (Table 2). This is an important aspect, considering that, at the time of sample collection, patients did not have pulmonary exacerbation, sinusitis, or other infection and were not on other medication such as the dual combination. Furthermore, we have observed a reduction in the CRP/albumin ratio, which, as described in the literature, is considered to be an independent predictor of mortality [35,36]. Again, we show that when comparing patients without and with liver disease (CFLD), we could see that reduction of CRP was less marked in the latter group, suggesting that, in subjects with liver disease, the inflammatory status could take longer to be reduced. Of note, as can be seen from Table 3, is that not all patients had levels of this marker over the reference value at the baseline.

We also observed a slight but significant increase in direct and total bilirubin as well as in AST, indicating that ETI might cause a mild stress/injury of the liver, even if only a few patients showed levels of these markers over the reference limits. Nevertheless, these markers should be constantly monitored during treatment.

As discussed previously, since CF patients have an abnormal lipid metabolism, studying the impact of ETI treatment on this aspect of the disease is of great importance. Here we show that ETI positively impacted lipid profiles in CF patients. To evaluate if the increment of lipid biomarkers and other parameters were dependent on the diet regimen, we compared the nutritional data at baseline and after one year of ETI therapy. The differences between diet composition at baseline and after one year of ETI therapy were not statistically significant. Therefore, we hypothesize that the changes in lipid biomarkers, observed after one year of therapy, were not dependent on the diet composition. Unfortunately, an exhaustive diet regimen was obtained only for 11 patients (55%) and this represents a study limitation.

We also evaluated the correlations between diet composition and anthropometric/biochemical parameters. In particular, we found inverse correlations of diet MuFA versus serum LDL, non-HDL cholesterol, and LDL/HDL ratio before the start of ETI therapy, and inverse correlations of diet carbohydrates versus the same lipid biomarkers after one year of ETI therapy. We also observed a positive correlation between the intake of protein and serum albumin after treatment. Although we have seen significant correlations between nutritional and anthropometric/biochemical data, we think that these results are not dietary dependent. However, as we have already mentioned, since the number of patients is limited, these results should be confirmed in a larger cohort.

Furthermore, we studied the variability of some biomarkers, in particular, albumin and non-HDL cholesterol. Since we observed a spread in the data distribution of these two biomarkers, we decided to evaluate the association between the variations of these parameters as a percentage and the genotypes of patients grouped into three clusters described in Table 1.

Interestingly, after one year of ETI therapy, the protein synthesis increased in all three groups, as shown by the serum albumin, and was not affected by the genotype. On the other hand, as far as lipid metabolism is concerned, the first genotype group, which includes patients with the most severe mutations (early truncation), did not show large variations, as suggested by non-HDL cholesterol, and the increases were significantly lower compared to the second and third groups.

## 5. Conclusions

In the present work, we examine for the first time the quantitative variations of liver function in relation to treatment with the triple combination of modulators (ETI) for the treatment of cystic fibrosis. In particular, despite the small number of patients studied, we show that there is a significant improvement in albumin synthesis/secretion compared to baseline values after one year of treatment. Furthermore, this result is also reinforced by the increased hepatic synthesis/secretion of lipoproteins, as evidenced by circulating non-HDL cholesterol. The increments of the latter biomarkers are influenced by the combination of the genotypes. These findings indicate that it is necessary to investigate these aspects in a greater number of patients and to consider non-HDL cholesterol as a useful indicator of hepatic lipoprotein synthesis for a better understanding of the effects of drugs.

## Figures and Tables

**Table 1:** Patient characteristics. Continuous normal data are reported as mean (SD). Categorical data are reported as frequency (percentage). CFLD: CF-associated liver disease; CFRD: CF-related diabetes; FEV1: forced expiratory volume in the 1st second.

Parameters	Values
Age, y. (SD)	31.9 (7.7)
Male, *n* (%)	8 (40)
Genotype group, *n* (%):	
First group	
Phe508del/G542X	3 (15)
Phe508del/R553X	1 (5)
Phe508del/G673X	1 (5)
Second group	
Phe508del/2183AA>G	2 (10)
Phe508del/R1158X	1 (5)
Phe508del/W1282X	1 (5)
Phe508del/4016insT	1 (5)
Phe508del/17a-17b-18del 9T/9T	1 (5)
Phe508del/*CFTR*dele14b17b	1 (5)
Phe508del/macro22-23del	1 (5)
Phe508del/1717-1G>A	1 (5)
Third group	
Phe508del/N1303K	5 (25)
Phe508del/G85E	1 (5)
*P. aeruginosa* colonization, *n* (%)	14 (70)
FEV1 (%)	31.4 (8.2)
CFLD, *n* (%)	12 (60)
CFRD, *n* (%)	12 (60)

**Table 2:** Comparison of anthropometric and biochemical parameters at baseline, and after 3 and 12 months of ETI therapy. Data are reported as median (IQR). ^a^: *p* < 0.05 vs. baseline, ^b^: *p* < 0.05 vs. 3 months. n.v.: normal values.

	n.v.	Baseline	3 Months	12 Months	*p* Value
Weight (kg)		53 (50–58.25)	56.6 (54.5–59.57) ^a^	58.2 (56.6–62.9) ^a,b^	0.021
BMI (kg/m^2^)		20 (19.20–21.75)	22 (20.62–23.75) ^a^	23.1 (21–24.8) ^a,b^	0.002
Glucose (mg/dL)	70–110	89 (79–101.2)	89.5 (71.25–102.5)	88 (86–109.7)	n.s.
HbA1C (%)		6.2 (5.92–7)	5.8 (5.35–6.97) ^a^	5.8 (5.2–6.2) ^a^	n.s.
Insulin		7.25 (4.10–16.53)	7.90 (5–38)	12.3 (5–31.8) ^a^	n.s.
Steatocrit (%)		4.1 (2.05–6.22)	3.1 (2.1–7.9)	2.25 (1.7–3.45)	n.s.
CRP (mg/dL)	0–0.5	0.71 (0.33–2.79)	0.33 (0.33–0.41) ^a^	0.30 (0.20–0.30) ^a^	<0.0001
CRP/Albumin ratio		0.17 (0.08–0.81)	0.08 (0.08–0.10) ^a^	0.07 (0.07–0.09) ^a,b^	0.0001
**Liver Parameters**					
AP (U/L)	40–150	109.5 (84–146)	133.5 (101.3–172.8)	119 (95–172)	n.s.
				119 (95–172)	
Dir Bil (mg/mL)	0–0.40	0.24 (0.16–0.34)	0.36 (0.26–0.44) ^a^	0.33 (0.26–0.62) ^a^	0.007
Total Bil (mg/mL)	0.20–1.20	0.50 (0.31–0.66)	0.78 (0.56–1.40) ^a^	0.95 (0.57–1.73) ^a,b^	0.003
AST (U/L)	0–34	20 (15–26.75)	25.50 (20.25–34.50) ^a^	27.5 (17.5–40.5) ^a^	0.047
ALT (U/L)	0–55	22.5 (11.7–28.7)	28 (22.50–53.25) ^a^	31.5 (20.25–47.75) ^a^	n.s.
yGT (U/L)	12–64	14.50 (10–20.75)	11.50 (9.25–45.25)	13 (10–29.75)	n.s.
Albumin (g/dL)	3.5–5.2	4.1 (3.7–4.4)	4.1 (4.0–4.3) ^a^	4.5 (4.4–4.7) ^a,b^	<0.0001
**Lipids**					
Total CHOL (mg/dl)	121–232	134 (101–145)	144 (114.8–152.8) ^a^	157.5 (135–167) ^a,b^	0.035
HDL CHOL (mg/dL)	>40	56 (45.50–70)	43 (41–53.75) ^a^	49 (44–66) ^b^	n.s.
LDL CHOL (mg/dL)	<115	47 (34–84)	79 (67–90) ^a^	91 (81–99) ^a,b^	0.0001
Trig (mg/dL)	<150	80.50 (69.25–106.5)	75 (66–82.50) ^a^	79 (62–103) ^b^	n.s.
Vit. A (μg/dL)	20–80	28.10 (21–36.15)	39.75 (27.45–57.38)	47.8 (39.2–61.8)	0.002
Vit. D (ng/mL)	30–100	29.40 (22–34.80)	25.25 (16.33–33.03)	27.6 (22.9–34.17)	n.s.
Vit. E (μg/dL)	500–1800	1181 (840.8–1481)	1193 (730.4–1495)	1316 (856.8–1803)	n.s.
non-HDL CHOL	<145	63.5 (45–104.8)	90.5 (76–105.8) ^a^	97 (90–114) ^a,b^	0.004
LDL/HDL ratio		0.69 (0.53–2.02)	1.79 (1.46–2.04) ^a^	1.93 (1.4–2.04) ^a^	0.005

Body Mass Index (BMI); Glycated hemoglobin (HbA1C); C-reactive protein (CRP); Alkaline phosphatase (AP); Direct Bilirubin (Dir Bil); Aspartate Aminotransferase (AST); Alanine aminotransferase (ALT); Gamma-glutamyltransferase (yGT); High-density lipoprotein (HDL) cholesterol (CHOL); Low-density lipoprotein (LDL); Triglycerides (Trig); Vitamin (Vit).

**Table 3:** CF patients with and without CFLD showing altered CRP and liver parameters at baseline and after 1 year of ETI therapy. ↑ Higher than normal values; ↓ lower than normal values.

	↑ CRP	↓ Albumin	↑ AP	↑ Dir. Bil.	↑ Total Bil.	↑ AST	↑ ALT	↑ yGT
CFLD (*n* = 12)		(Number of patients)						
Baseline	7	2	3	2	1	1	1	1
After 1 year	4	-	4	5	5	2	1	2
No CFLD (*n* = 8)								
Baseline	5	-	1	-	-	1	-	1
After 1 year	-	-	3	4	3	3	3	1

C-reactive protein (CRP); Alkaline phosphatase (AP); Direct Bilirubin (Dir Bil); Aspartate Ami-notransferase (AST); Alanine aminotransferase (ALT); Gamma-glutamyltransferase (yGT).

**Table 4:** Comparison between the nutritional data at baseline and 12 months (*n* = 11). Data are reported as mean (SD). Abbreviations: Carbohydrates (Carb), Cholesterol (Chol), Saturated fatty acid (SFA), Monounsaturated fatty acids (MuFA), Polyunsaturated fatty acids (PuFA).

Components	Baseline	12 Months	*p* Value
Kcal	2167 (472)	2131 (496)	0.66
Proteins (g)	102 (16)	97 (22)	0.56
Proteins (%)	19 (3)	18 (3)	0.53
Lipids (g)	80 (30)	85 (22)	0.42
Lipids (%)	33 (6)	37 (9)	0.29
Carb (g)	272 (61)	255 (70)	0.33
Carb (%)	50 (6)	48 (12)	0.33
Fibers (g)	18 (3)	20 (6)	0.10
Calcium (mg)	771 (245)	798 (219)	0.93
Chol (mg)	224 (59)	237 (67)	0.92
SFA (g)	10 (7)	10 (4)	0.72
SFA (%)	4.1 (1.8)	4.2 (1.6)	0.86
MuFA (g)	35 (11)	31 (7)	0.13
MuFA (%)	14 (3)	13 (3)	0.11
PuFA (g)	6.0 (2.8)	5.7 (1.8)	0.76
PuFA %	2.5 (0.7)	2.4 (0.7)	0.77

## Data Availability

The data are available upon request from the corresponding author.

## Supplementary Materials

The following supporting information can be downloaded at: https://www.mdpi.com/article/10.3390/jcm11236900/s1, Table S1: Spearman correlation analysis between bromatological and biochemical data at baseline; Table S2: Spearman correlation analysis between bromatological and biochemical data at 12 months; Table S3: Protein and metabolic changes after one year of ETI treatment in patients with CF carrying Minimal Function (MF) heterozygosity with Phe508del mutation.
