# Supplementary material for: Cystic Fibrosis Patients with F508del/Minimal Function Genotype: Laboratory and Nutritional Evaluations after One Year of Elexacaftor/Tezacaftor/Ivacaftor Treatment
Source: J Clin Med. 2022 Nov 22;11(23):6900. doi: 10.3390/jcm11236900 (PMC9735556; doi:10.3390/jcm11236900)
Supplement: Supplementary file 1 [file jcm-11-06900-s001.zip › jcm-1995284-supplementary.pdf]

Table S1. Spearman correlation analysis between bromatological and biochemical data at baseline

		BMI (kg/m^2)	Vit A (μg/dL)	Album (g/dL)	CHOL (mg/dL)	LDL (mg/dL)	CHOL/ HDL ratio	no-HDL CHOL (mg/dL)	LDL/HDL ratio
**Kcal/day**	r_s	-0.198	0.151	-0.357	-0.536	-0.364	0.227	-0.382	0.073
	p	0.559	0.699	0.281	0.089	0.272	0.502	0.247	0.832
**Protein (g)**	r_s	-0.032	0.088	-0.002	-0.009	-0.005	0.200	-0.009	0.091
	p	0.925	0.821	0.995	0.979	0.989	0.555	0.979	0.790
**Lipids (g)**	r_s	-0.284	-0.176	-0.266	-0.551	-0.465	-0.205	-0.538	-0.196
	p	0.397	0.651	0.429	0.079	0.150	0.545	0.088	0.564
**Carb (g)**	r_s	-0.268	0.756	-0.163	-0.451	-0.105	0.497	-0.164	0.392
	p	0.426	**0.018**	0.632	0.164	0.759	0.120	0.630	0.233
**Fibers (g)**	r_s	0.196	0.662	-0.095	-0.111	0.005	0.346	0.041	0.230
	p	0.563	0.052	0.781	0.746	0.989	0.298	0.904	0.495
**Calcium (mg)**	r_s	0.143	0.268	-0.279	-0.591	-0.491	0.073	-0.491	-0.064
	p	0.675	0.486	0.406	0.056	0.125	0.832	0.125	0.853
**Chol (mg)**	r_s	-0.074	-0.201	-0.151	-0.445	-0.555	-0.282	-0.536	-0.318
	p	0.829	0.604	0.658	0.170	0.077	0.401	0.089	0.340
**SFA (g)**	r_s	-0.320	-0.272	0.130	-0.221	-0.355	-0.571	-0.396	-0.424
	p	0.337	0.478	0.703	0.513	0.284	0.066	0.228	0.194
**MuFA (g)**	r_s	0.092	-0.055	-0.041	-0.350	-0.613	-0.805	-0.628	-0.676
	p	0.787	0.888	0.905	0.292	**0.045**	**0.003**	**0.039**	**0.023**
**PuFA (g)**	r_s	-0.189	0.277	0.420	-0.292	-0.359	-0.541	-0.441	-0.426
	p	0.577	0.471	0.199	0.383	0.278	0.085	0.175	0.191

Table S2. Spearman correlation analysis between bromatological and biochemical data at 12 months

		BMI (kg/m^2)	Vit A (µg/dL)	Album (g/dL)	CHOL (mg/dL)	LDL (mg/dL)	CHOL/ HDL ratio	no-HDL CHOL (mg/dL)	LDL/HDL ratio
**Kcal/day**	r_s	-0.437	-0.400	0.571	-0.101	-0.164	-0.591	-0.487	-0.638
	p	0.179	0.286	0.066	0.768	0.631	0.056	0.128	**0.035**
**Protein (g)**	r_s	-0.513	-0.251	0.664	0.354	0.096	-0.224	-0.146	-0.137
	p	0.107	0.515	**0.026**	0.285	0.779	0.508	0.667	0.687
**Lipids (g)**	r_s	-0.308	-0.583	0.350	-0.128	-0.182	-0.373	-0.415	-0.487
	p	0.357	0.099	0.291	0.707	0.593	0.259	0.205	0.128
**Carb (g)**	r_s	-0.363	-0.083	0.470	-0.252	-0.382	-0.773	-0.642	-0.797
	p	0.272	0.831	0.145	0.455	0.247	**0.005**	**0.033**	**0.003**
**Fibers (g)**	r_s	-0.023	-0.100	-0.076	-0.218	-0.173	0.387	0.009	0.409
	p	0.946	0.798	0.824	0.520	0.611	0.239	0.979	0.212
**Calcium (mg)**	r_s	-0.525	-0.234	-0.152	-0.216	-0.282	0.068	-0.137	0.023
	p	0.097	0.544	0.655	0.524	0.400	0.842	0.688	0.947
**Chol (mg)**	r_s	-0.830	-0.519	0.453	0.119	-0.146	-0.401	-0.326	-0.438
	p	**0.002**	0.152	0.162	0.727	0.669	0.222	0.327	0.177
**SFA (g)**	r_s	-0.567	-0.667	0.292	0.005	-0.014	-0.197	-0.144	-0.229
	p	0.069	0.050	0.383	0.989	0.968	0.562	0.672	0.498
**MuFA (g)**	r_s	-0.125	-0.815	0.193	0.118	0.394	0.430	0.369	0.431
	p	0.714	0.007	0.571	0.731	0.231	0.187	0.264	0.185
**PuFA (g)**	r_s	0.131	-0.009	0.257	-0.014	0.157	-0.147	0.122	-0.049
	p	0.702	0.983	0.446	0.968	0.645	0.665	0.720	0.887

Table S3. Protein and metabolic changes after one year of ETI treatment in patients with CF carrying Minimal Function (MF) heterozygosity with Phe508del mutation

Genotype group	Class of defect	Protein changes	Albumin changes (%)		non-HDL CHOL changes (%)	
			average (SD)	min-max	average (SD)	min-max
First group (n = 5)	I	early stop truncation	15 (10)	0 – 24	18 (51)	-28 – 105
Second group (n = 9)	I	late stop truncation and other MF defects	19 (5)	5 – 29	59 (40) *	4 – 129
Third group (n = 6)	II	Other not responsive MF defects [a]	11 (10)	0 – 29	69 (46) *	8 – 159

*p < 0.05 vs first group. [a] Not responsive to ivacaftor or tezacaftor. ETI: elexacaftor/tezacaftor/ivacaftor
